# mbs: modifying Hudson's ms software to generate samples of DNA sequences with a biallelic site under selection

**DOI:** 10.1186/1471-2105-10-166

**Published:** 2009-05-30

**Authors:** Kosuke M Teshima, Hideki Innan

**Affiliations:** 1 Hayama Center for Advanced Studies, Graduate University for Advanced Studies, Hayama, Kanagawa 240-0193, Japan

## Abstract

**Background:**

The pattern of single nucleotide polymorphisms, or SNPs, contains a tremendous amount of information with respect to the mechanisms of the micro-evolutionary process of a species. The inference of the roles of these mechanisms, including natural selection, relies heavily on computer simulations. A coalescent simulation is extremely powerful in generating a large number of samples of DNA sequences from a population (species) when all mutations are neutral, and Hudson's **ms **software is frequently used for this purpose.

However, it has been difficult to incorporate natural selection into the coalescent framework.

**Results:**

We herein present a software application to generate samples of DNA sequences when there is a biallelic site targeted by selection. This software application, referred to as **mbs**, is developed by modifying Hudson's **ms**. The **mbs **software is so flexible that it can incorporate any arbitrary histories of population size changes and any mode of selection as long as selection is operating on a biallelic site.

**Conclusion:**

**mbs **provides opportunities to investigate the effect of any mode of selection on the pattern of SNPs under various demography.

## Background

The coalescent provides a very efficient simulation tool for generating DNA samples drawn from populations [1,2]. Hudson's software, **ms**, is widely used in population genetics largely because of its flexibility [2]. **ms **can generate patterns of DNA polymorphism under the infinite-site model with a complicated demographic history, given that all mutations are neutral. **ms **is frequently used for estimating demographic and mutational parameters (including point mutation and recombination rates) and for testing for natural selection.

However, provided that a number of genes in a genome are subject to selection, understanding how selection affects the pattern of DNA polymorphism is very important in population genetics (e.g., [3]). Incorporating selection into the coalescent has been a challenging problem, and one approach has been to consider a biallelic-structure with the original and derived allelic classes [4]. The frequencies of the two allelic classes can change over time, and once their historical trajectory is given, the coalescent algorithm can trace the ancestral lineages of sampled chromosomes backward in time conditional on the trajectory. A simple application of this idea is to perform a selective sweep [5], in which the trajectory of a beneficial allele in its quick fixation process is given in a deterministic form.

In addition, it is possible to apply this idea to more complex modes of selection. The most important point is that the coalescent works as long as a trajectory of the two allelic classes is given. The trajectory can be obtained by any method, including theory and simulation. This flexibility allows us to incorporate any mode of selection together with the effect of random genetic drift at the biallelic site. The changes of the past population sizes can also be simultaneously considered. However, modification of the standard coalescent algorithm to incorporate these complexities is relatively difficult and feasible for only a limited number of specialists [6,7].

Here, we provide a very user-friendly software application to generate a biallelic sample of DNA sequences (called **mbs**), which incorporates any change in the trajectory of allelic frequency and population size (Additional file 1). The software has inline commands and an output form similar to those of Hudson's **ms **[2]. The allelic frequency trajectory and population size changes must be prepared (either theoretically or by simulation, or even arbitrarily) and stored in an input file before running **mbs**. This flexibility enables users to simulate patterns of DNA polymorphism in any situation, as long as selection works at a single biallelic site. The software can be widely used for advanced purposes, including simultaneous inferences of selection and demography.

## Implementation

The software application assumes the Wright-Fisher model in a finite population. Following Hudson's **ms **[2], the standard coalescent assumptions are used to simulate a random genealogical history of a recombining chromosome and to place random mutations on the chromosome. The basic neutral parameters, (*i.e.*, population mutation and recombination rates) are given by per-site rates, 4*N*_0_*μ *and 4*N*_0_*r*, respectively, where *N*_0 _is the current population size and *μ *and *r *are the mutation and recombination rates, respectively, per bp per generation. Multiple mutations at a single site are allowed. In addition, we assume that there is a single biallelic site targeted by selection in the simulated region, which consists of a finite number of neutral sites. At the selected site, all chromosomes have either of the two states, 0 or 1, representing the original and derived allelic classes, respectively. The treatment of these two allelic classes is similar to that of two subpopulations. In brief, coalescent events are limited to chromosomes within the same allelic class. The time to the next coalescent event (backward in time) depends on the population size (or frequency) of the allelic class. A mutation at the selected site will change the allelic class. In addition, migration of a partial segment occurs through recombination between the two allelic classes.

## Results and Discussion

### INPUT

**Figure 1:**
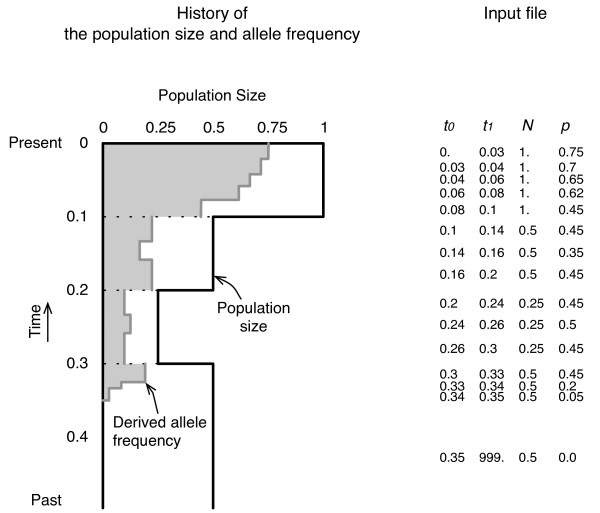
**An example of the allele frequency and demographic change**. Illustration of an example of the allele frequency and demographic changes (left), and their input format (right).

The **mbs **software requires an input file, which includes the past history of the allelic frequency and population size. In **mbs**, any change in the population size and allele frequency is treated as a stepwise change, as illustrated in Figure 1. The population size is scaled in units of the current population size, *N*_0_, and time is measured in units of 4*N*_0 _generations. Each line in the input file has four values, namely, the beginning and end times of the phase (from *t*_0 _to *t*_1_), the population size (*N*), and the derived allele frequency (*p*), where *t*_1 _in the last line must be 999, which technically denotes infinity. If the time intervals are set to be small, the trajectory and population size changes can be nearly continuous with an increased computational time.

To generate samples conditional on input files, **mbs **requires a command line similar to that of **ms**. An example is:

./mbs nsam -t 0.01 -r 0.01 -s 1000 250 -f 2 5 traj

This command generates a sample of chromosomes of size *nsam*, which will be randomly assigned to either the ancestral or derived allelic classes according to their current frequencies. Here, -t and -r specify the population mutation and recombination rates per bp, which are set to be 4*N*_0 _*μ *= 4*N*_0_*r *= 0.01 in this example. The integers following the '-s' switch represent the number of sites in the simulated region and the position of the selected site, respectively. The command line argument '-f 2 5 traj' means that the software performs five replications of the simulation for two independent histories of *N *and *p *(for a total of 10 replications), which are stored in separate files named "traj_0.dat" and "traj_1.dat". In general, a single run of **mbs **accepts a finite number (say, *k*) of input files named "traj_0.dat", "traj_1.dat", ⋯, "traj_k-1.dat".

### OUTPUT

The output of **mbs **is almost identical to that of Hudson's **ms **with a few modifications. For each replication, the simulated pattern of polymorphism is output as follows. The first line indicates that this is the result of the first replication with the first trajectory file ("traj_0.dat"). The allelic status of the selected site (a: ancestral, d: derived) for the sample is also given. The second line gives *S*, i.e., the number of neutral polymorphic sites, the positions of which are provided in the next line. The following *nsam *lines are for the polymorphism information of the sampled chromosomes. Each line consists of a string of 0 s and 1 s, representing the allelic status at the *S *neutral polymorphic sites listed above. The following is an example:

//0-1 allele: a a d d

segsites: 14

positions: 76 205 213 240 251 335 370 506 599 698 749 948 984 997

11001000000101

11001010000101

00110101101000

00110101011010

where the sample size is *nsam *= 4, the first two samples of which are assigned to the ancestral allelic class and the final two samples of which are assigned to the derived allelic class.

### OTHER OPTIONS

There are other possible complications, which are briefly described below. Detailed documentation is available at the web site indicated below. We also provide a simple simulation program to generate trajectories of allele frequencies under typical modes of selection, including directional selection with arbitrary dominance (including selective sweep) and overdominant selection.

#### Mutation model

While an output consists of sequences with two allelic states, 0 and 1, with the default setting, an optional command allows the creation of sequences with four allelic states, 0, 1, 2, and 3, which represent the four nucleotides, A, T, G, and C.

#### Recombination rate heterogeneity

The default setting assumes a uniform distribution of recombination over the simulated region, but recombination hot spots or any kind of distribution of recombination rate can be incorporated. In this case, another input file is required.

## Conclusion

We have presented a software application, **mbs**, to generate samples of DNA sequences when there is a biallelic site targeted by selection. **mbs **was developed by modifying commonly used Hudson's **ms **software, so that it has inline commands and an output form similar to those of **ms**. The **mbs **software is so flexible that it can incorporate any arbitrary histories of population size changes and any mode of selection. This provides opportunities to investigate the effect of any mode of selection on the pattern of SNPs under various demography.

## Availability and requirements

• **Project name**: mbs

• **Project home page**: 

• **Operating system**: Platform independent

• **Programming language**: C

• **Other requirements**: none

• **License**: none

• **Any restrictions on use by non-academics**: none

## Authors' contributions

KMT and HI conceived the study and wrote the manuscript. KMT implemented the code. Both authors have read and approved the manuscript.

## Supplementary Material

Additional file 1 **mbs source code and readme file**. The compressed source code and the readme file for *mbs*. Click here for file
