# Supplementary material for: mbs: modifying Hudson's ms software to generate samples of DNA sequences with a biallelic site under selection
Source: BMC Bioinformatics. 2009 May 30;10:166. doi: 10.1186/1471-2105-10-166 (PMC2693440; doi:10.1186/1471-2105-10-166)
Supplement: Additional file 1 — mbs source code and readme file. The compressed source code and the readme file for mbs. [file 1471-2105-10-166-S1.zip › SourceFile_TeshimaInnan/mbs_TeshimaInnan.pdf]

mbs: modifying Hudson's ms software to generate samples of DNA sequences with a biallelic site under selection

Kosuke M. Teshima*[1] and Hideki Innan[1]

[1]The Graduate University for Advanced Studies, Hayama, Kanagawa 240-0193, Japan

Email: Kosuke M. Teshima*- kteshima@soken.ac.jp; Hideki Innan - innan_hideki@soken.ac.jp;

*Corresponding author

Abstract

Background: The pattern of single nucleotide polymorphisms, or SNPs, contains a tremendous amount of information with respect to the mechanisms of the micro-evolutionary process of a species. The inference of the roles of these mechanisms, including natural selection, relies heavily on computer simulations. A coalescent simulation is extremely powerful in generating a large number of samples of DNA sequences from a population (species) when all mutations are neutral, and Hudson's **ms** software is frequently used for this purpose. However, it has been difficult to incorporate natural selection into the coalescent framework.

Results: We herein present a software application to generate samples of DNA sequences when there is a biallelic site targeted by selection. This software application, referred to as **mbs**, is developed by modifying Hudson's **ms**. The **mbs** software is so flexible that it can incorporate any arbitrary histories of population size changes and any mode of selection as long as selection is operating on a biallelic site.

Conclusions: **mbs** provides opportunities to investigate the effect of any mode of selection on the pattern of SNPs under various demography.

Background

The coalescent provides a very efficient simulation tool for generating DNA samples drawn from populations [1, 2]. Hudson's software, **ms**, is widely used in population genetics largely because of its flexibility [2]. **ms** can generate patterns of DNA polymorphism under the infinite-site model with a complicated demographic history, given that all mutations are neutral. **ms** is frequently used for estimating demographic and mutational parameters (including point mutation and recombination rates) and for testing for natural selection.

However, provided that a number of genes in a genome are subject to selection, understanding how selection affects the pattern of DNA polymorphism is very important in population genetics (e.g., [3]). Incorporating selection into the coalescent has been a challenging problem, and one approach has been to consider a biallelic-structure with the original and derived allelic classes [4]. The frequencies of the two allelic classes can change over time, and once their historical trajectory is given, the coalescent algorithm can trace the ancestral lineages of sampled chromosomes backward in time conditional on the trajectory. A simple application of this idea is to perform a selective sweep [5], in which the trajectory of a beneficial allele in its quick fixation process is given in a deterministic form.

In addition, it is possible to apply this idea to more complex modes of selection. The most important point is that the coalescent works as long as a trajectory of the two allelic classes is given. The trajectory can be obtained by any method, including theory and simulation. This flexibility allows us to incorporate any mode of selection together with the effect of random genetic drift at the biallelic site. The changes of the past population sizes can also be simultaneously considered. However, modification of the standard coalescent algorithm to incorporate these complexities is relatively difficult and feasible for only a limited number of specialists [6, 7].

Here, we provide a very user-friendly software application to generate a biallelic sample of DNA sequences (called **mbs**), which incorporates any change in the trajectory of allelic frequency and population size. The software has inline commands and an output form similar to those of Hudson's **ms** [2]. The allelic frequency trajectory and population size changes must be prepared (either theoretically or by simulation, or even arbitrarily) and stored in an input file before running **mbs**. This flexibility enables users to simulate patterns of DNA polymorphism in any situation, as long as selection works at a single biallelic site. The software can be widely used for advanced purposes, including simultaneous inferences of selection and demography.

Implementation

The software application assumes the Wright-Fisher model in a finite population. Following Hudson's **ms** [2], the standard coalescent assumptions are used to simulate a random genealogical history of a recombining chromosome and to place random mutations on the chromosome. The basic neutral parameters, (*i.e.,* population mutation and recombination rates) are given by per-site rates, $4N_0\mu$ and $4N_0r$, respectively, where N_0 is the current population size and μ and r are the mutation and recombination rates, respectively, per bp per generation. Multiple mutations at a single site are allowed. In addition, we assume that there is a single biallelic site targeted by selection in the simulated region, which consists of a finite number of neutral sites. At the selected site, all chromosomes have either of the two states, 0 or 1, representing the original and derived allelic classes, respectively. The treatment of these two allelic classes is similar to that of two subpopulations. In brief, coalescent events are limited to chromosomes within the same allelic class. The time to the next coalescent event (backward in time) depends on the population size (or frequency) of the allelic class. A mutation at the selected site will change the allelic class. In addition, migration of a partial segment occurs through recombination between the two allelic classes.

Results

INPUT

The **mbs** software requires an input file, which includes the past history of the allelic frequency and population size. In **mbs**, any change in the population size and allele frequency is treated as a stepwise change, as illustrated in Figure 1. The population size is scaled in units of the current population size, N_0, and time is measured in units of $4N_0$ generations. Each line in the input file has four values, namely, the beginning and end times of the phase (from t_0 to t_1), the population size (N), and the derived allele frequency (p), where t_1 in the last line must be 999, which technically denotes infinity. If the time intervals are set to be small, the trajectory and population size changes can be nearly continuous with an increased computational time.

To generate samples conditional on input files, **mbs** requires a command line similar to that of **ms**. An example is:

```
./mbs nsam -t 0.01 -r 0.01 -s 1000 250 -f 2 5 traj
```

This command generates a sample of chromosomes of size *nsam*, which will be randomly assigned to either the ancestral or derived allelic classes according to their current frequencies. Here, -t and -r specify the

population mutation and recombination rates per bp, which are set to be $4N_0\mu = 4N_0r = 0.01$ in this example. The integers following the '-s' switch represent the number of sites in the simulated region and the position of the selected site, respectively. The command line argument '-f 2 5 traj' means that the software performs five replications of the simulation for two independent histories of N and p (for a total of 10 replications), which are stored in separate files named "traj_0.dat" and "traj_1.dat". In general, a single run of **mbs** accepts a finite number (say, k) of input files named "traj_0.dat", "traj_1.dat", $\cdots$, "traj_k-1.dat".

OUTPUT

The output of **mbs** is almost identical to that of Hudson's **ms** with a few modifications. For each replication, the simulated pattern of polymorphism is output as follows. The first line indicates that this is the result of the first replication with the first trajectory file ("traj_0.dat"). The allelic status of the selected site (`a`: ancestral, `d`: derived) for the sample is also given. The second line gives S, i.e., the number of neutral polymorphic sites, the positions of which are provided in the next line. The following *nsam* lines are for the polymorphism information of the sampled chromosomes. Each line consists of a string of 0s and 1s, representing the allelic status at the S neutral polymorphic sites listed above. The following is an example:

```
//0-1   allele: a a d d
segsites: 14
positions: 76 205 213 240 251 335 370 506 599 698 749 948 984 997
11001000000101
11001010000101
00110101101000
00110101011010
```

where the sample size is $nsam = 4$, the first two samples of which are assigned to the ancestral allelic class and the final two samples of which are assigned to the derived allelic class.

OTHER OPTIONS

There are other possible complications, which are briefly described below. Detailed documentation is available at the web site indicated below. We also provide a simple simulation program to generate

trajectories of allele frequencies under typical modes of selection, including directional selection with arbitrary dominance (including selective sweep) and overdominant selection.

Mutation model: While an output consists of sequences with two allelic states, 0 and 1, with the default setting, an optional command allows the creation of sequences with four allelic states, 0, 1, 2, and 3, which represent the four nucleotides, A, T, G, and C.

Recombination rate heterogeneity: The default setting assumes a uniform distribution of recombination over the simulated region, but recombination hot spots or any kind of distribution of recombination rate can be incorporated. In this case, another input file is required.

Conclusions

We have presented a software application, **mbs**, to generate samples of DNA sequences when there is a biallelic site targeted by selection. **mbs** was developed by modifying commonly used Hudson's **ms** software, so that it has inline commands and an output form similar to those of **ms**. The **mbs** software is so flexible that it can incorporate any arbitrary histories of population size changes and any mode of selection. This provides opportunities to investigate the effect of any mode of selection on the pattern of SNPs under various demography.

Availability and requirement

- **Project name:** mbs
- **Project home page:** http://www.sendou.soken.ac.jp/esb/innan/InnanLab/Index_En.html
- **Operating system:** Platform independent
- **Programming language:** C
- **Other requirements:** none
- **License:** none
- **Any restrictions on use by non-academics:** none

Authors contributions

KMT and HI conceived the study and wrote the manuscript. KMT implemented the code. Both authors have read and approved the manuscript.

Acknowledgements

The present study was supported by grants to HI from the Graduate University for Advanced Studies and from the Japan Society for the Promotion of Science.

References

1. Hudson R: **Gene genealogies and the coalescent process.** In *Oxford Surveys in Evolutionary Biology, Volume 7.* Edited by Futuyma D, Antonovics J, Oxford University Press 1990:1–44.
2. Hudson R: **Generating samples under a Wright-Fisher neutral model of genetic variation.** *Bioinformatics.* 2002, **18**:337–338.
3. Nielsen R, Hellmann I, Hubisz M, Bustamante C, Clark AG: **Recent and ongoing selection in the human genome.** *Nat. Rev. Genet.* 2007, **8**:857–68.
4. Hudson R, Kaplan N: **The coalescent process in models with selection and recombination.** *Genetics* 1988, **120**:831–40.
5. Bravermann JM, Hudson RR, Kaplan NL, Langley CH, Stephan W: **The hitchhiking effect on the site frequency spectrum of DNA polymorphisms.** *Genetics* 1995, **140**:783–796.
6. Kim Y, Stephan W: **Detecting a local signature of genetic hitchhiking along a recombining chromosome.** *Genetics* 2002, **160**:765–77.
7. Teshima K, Coop G, Przeworski M: **How reliable are empirical genomic scans for selective sweeps?** *Genome Res* 2006, **16**:702–12.

Figures

Figure 1 - An example of the allele frequency and demographic change

Illustration of an example of the allele frequency and demographic changes (left), and their input format (right).

Additional Files

Additional file 1 — Additional file 1 contains the compressed source code and the Readme file for *mbs*.

Click here for Additional file 1

[http://www.sendou.soken.ac.jp/esb/innan/InnanLab/Index_En.html/BMC_TeshimaInnan2009.tar.gz]
