# Supplementary material for: mbs: modifying Hudson's ms software to generate samples of DNA sequences with a biallelic site under selection
Source: BMC Bioinformatics. 2009 May 30;10:166. doi: 10.1186/1471-2105-10-166 (PMC2693440; doi:10.1186/1471-2105-10-166)
Supplement: Additional file 1 — mbs source code and readme file. The compressed source code and the readme file for mbs. [file 1471-2105-10-166-S1.zip › SourceFile_TeshimaInnan/readme.pdf]

mbs : readme file

Kosuke M. Teshima and Hideki Innan

kteshima@soken.ac.jp & innan_hideki@soken.ac.jp

The Graduate University for Advanced Studies, Hayama, Kanagawa 240-0193, Japan.

March 5, 2009

1 mbs

mbs is a simulation program to generate patterns of single nucleotide polymorphism (SNP) data in a region that is linked to a biallelic site targeted by selection. The software was developed by modifying the commonly used Hudson's ms simulator (Hudson, 1990). mbs is flexible so that it can incorporate any mode of selection and any change of the population size simultaneously. The software runs coalescent simulations conditional on the histories (trajectories) of allele frequency and population size, which should be stored in text files prior to running mbs .

1.1 Download and compilation

All files are included in the `source_TeshimaInnan.tar.gz` file, which can be downloaded at `http://www.sendou.soken.ac.jp/esb/innan/InnanLab`. The source code of the program is written in C and this program is intended to be run on UNIX, or a UNIX-like operating systems, such as Linux or Mac OS X.

Download the tar file onto your machine and extract it with: `tar -xzvf source_TeshimaInnan.tar.gz`. After extraction, change the directory by typing: `cd SourceFile_TeshimaInnan`. Then, compile the program by typing:

```
gcc -o mbs main.c ancestral_graph.c rand_mt32.c -lm
```

1.2 The basic command line

The following command line shows the simplest usage of mbs .

`./mbs` *nsam* `-t` $4N_0\mu$ `-r` $4N_0r$ `-s` *nsites selpos*
`-f` *nfile nrep filename*

nsam is the sample size. $4N_0\mu$ after the '-t' switch is the population mutation parameter per site, where N_0 is the current population size and μ is the mutation rate per site. $4N_0r$ after the '-r' switch is the population recombination parameter, where r is the recombination rate per site. Note that the per-bp

rates are given for `mbs`, while Hudson's `ms` requires the per-region rates. The two numbers after the '-s' switch represent the length of the simulated region in bp and the position of the selected site. The first site of the simulated region is set to be 0, and the position of the target site of selection can take both negative and positive values. This means that the location of the target site can be inside or outside of the simulated region.

The arguments after the '-f' switch, *nfile nrep filename*, specify the number of trajectory files, the number of replications for each trajectory file, and the initial part of the trajectory file name. For example, if '-f 2 5 traj' is given, `mbs` performs five replications of the simulation for two trajectory files (for a total of 10 replications), which must be named `traj_0.dat` and `traj_1.dat`. In general, a single run of `mbs` accepts a finite number (say, k) of input trajectory files named `traj_0.dat`, `traj_1.dat`, $\cdots$, `traj_k-1.dat`. The details of the trajectory file are explained in Section 1.3.

The following table is a summary of arguments that must be specified in order to run `mbs`.

switch	argument	
	nsam	number of chromosomes in the sample.
-t	$4N_0\mu$	population mutation parameter per site.
-r	$4N_0r$	population recombination parameter per site.
-s	*nsites*	length of the simulated region.
	selpos	position of the target site of selection relative to the first site of the simulated region.
-f	*nfile*	number of trajectory files.
	nrep	number of replications for each trajectory.
	filename	initial part of the name of the trajectory files.

The following is an example command line:

```
./mbs 4 -t 0.01 -r 0.01 -s 1000 250 -f 2 5 traj
```

In this case, the program will output SNP data in a 1,000 bp region, where the target site of selection is at position 250 bp. The sample size is $nsam = 4$, and $4N_0\mu = 4N_0r = 0.01$ are given. Five replications are performed for two trajectory files, `traj_0.dat` and `traj_1.dat`. Two example trajectory files, named `traj_0.dat` and `traj_1.dat`, are already included in the same directory, so that typing the above command line should will demonstrate the process. The output of the result is explained in Section 1.4.

1.3 The input file

The trajectory files should include the joint history of the allele frequency and population size. In `mbs`, any change in population size or allele frequency is treated as a stepwise change. Figure 1 illustrates an example history of the population (left) together with a corresponding input file, which is exactly the same as the `traj_0.dat` file in the `SourceFile_TeshimaInnan` directory.

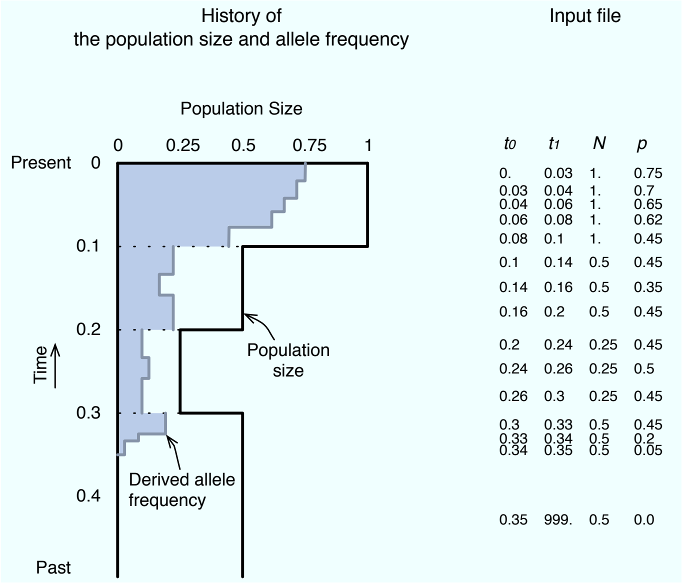

Figure 1

Time is measured toward the past in units of $4N_0$ generations, which is four times the current population size. In the input file, the population size is given in units of the current population size, N_0. This example assumes that, toward the past, the population size is half the current population size at $t = 0.1$, one-quarter the current population size at $t = 0.2$, and half the current population size at $t = 0.3$. The current frequency of the derived allele is $p = 0.75$. The allele originated upon the initial mutation that occurred at $t = 0.35$.

Each line in the input file consists of four values, namely, the beginning and end times of the phase (from t_0 to t_1), the population size (N), and the derived allele frequency (p). If time intervals are set to be small, then the trajectory and population size changes become almost continuous with increased computational time.

There are two rules. (i) t_1 in the last line must be 999, which technically denotes infinity. If t_1 is set to 999, then N and p are constant until ancestral lineages reach the most recent common ancestor. Conversely, t_1 for the oldest phase must be 999. (ii) p may become 1 multiple times, but can become 0, which indicates the origin of the derived allelic class, only once. Once p becomes 0, there can be no more allele frequency changes ($p = 0$).

Other examples

`mbs` accepts any trajectory that could represent a realization of any mode of selection. Here, we prepare two more trajectory files, representing balancing selection and selective sweep with bottleneck, named `trajBS_0.dat` and

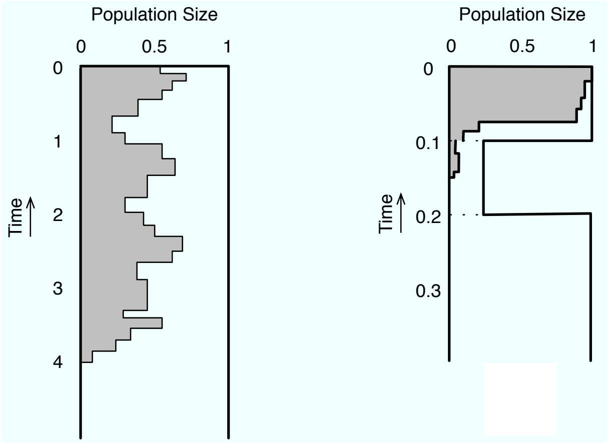

Figure 2: Illustrations of examples of trajectories for balancing selection (left), and selective sweep with bottleneck (right), in the trajectory files `trajBS_0.dat` and `trajSW_0.dat`, respectively.

`trajSW_0.dat`, respectively. The two example trajectories are illustrated in Figure 2. To use these files, type in one of their filenames as the argument after the '-f' switch, e.g., '-f 1 5 trajBS'. (The files must be copied in the working directory, `SourceFile_TeshimaInnan`, by typing
`cp ./Trajectory/eg_BS/trajBS_0.dat ..`)

1.4 The output

The example command in Section 1.2

```
./mbs 4 -t 0.01 -r 0.01 -s 1000 250 -f 2 5 traj
```

produces an output of $2 \times 5 = 10$ patterns of SNPs. The output format of `mbs` is very similar to that of `ms`, as described by the following example:

```
//0-1   allele: a a d d
segsites: 19
positions: 25 72 96 110 158 193 209 230 231 254 286 361 364 399 507 512 550 743 922
0111011011001100101
0111011011000100111
1000000100110011000
0111111011000100101

//0-2   allele: a a d d
segsites: 10
positions: 226 433 509 596 676 683 936 958 962 984
0001000101
0110100010
1000001000
```

```
0000010101
...
```

This example shows the results of the first two replications of the example command. The output of each replication starts with a line with '`//`', followed by two numbers. For example, '`//`i-j' means that the sample is the result of the jth replication generated conditional on the ith trajectory file, *i.e.*, `traj_`i`.dat`. The allelic states at the selected site (*a*: ancestral, *d*: derived) for the *nsum* chromosomes are listed in the same line. The next two lines provide the number of SNPs ('`segsites:`') and their positions ('`positions:`'). The haplotypes of each of the sampled chromosomes are given in the following *nsam* lines. Each line consists of a string of 0s and 1s, 0 indicates that the site is in an ancestral state and 1 indicates that the site is in a derived state.

1.5 Options

There is some flexibility in the mutation mode and local recombination variation.

Mutation:

The finite site mutation model is assumed in `mbs`. Each site can take the binary-state, '0' or '1', in the default setting, but this can be changed to the quaternary-state, '0', '1', '2', or '3'. To change to the quaternary-state model, add '-n 4' in a command line.

Recombination:

In the default setting, recombination occurs at equal probability r per bp. Incorporation of recombination hotspots requires an additional argument with the '-h' switch that calls a text file that specifies the positions of hotspots and the recombination rates at those hotspots. For example, type

```
./mbs 20 -t 0.01 -r 0.01 -s 10000 5000 -f 1 10 traj
                                 -h hotspot_example.dat
```

Then, `mbs` reads the file named `hotspot_example.dat`. An example of the hotspot input file is shown below.

— `hotspot_example.dat` —

```
100     5
101     5
102     5
103     5
255     7
256     7
257     7
```

The content of the hotspot input file consists of two columns. The first column is for the positions of hotspots, which must be an integer number, say x ($0 \leq x \leq nsites - 2$). The second column is for the recombination rate between positions x and $x + 1$, which is given by the relative rate as $4N_0r$. The relative rate must be a positive integer (≥ 1).

2 Simulation programs to make trajectory files

In addition, we provide two simple simulation programs, `forwardtraj` and `backwardtraj`, to generate trajectory files in the subdirectory `Trajectory`. These programs are to perform forward and backward simulations using a simplified pseudo-random-sampling method (Kimura and Takahata, 1983).

Let us assign *a* to the ancestral allele and *d* to the derived allele. The fitnesses for genotypes *aa*, *ad*, and *dd* are given by 1, $1+hs$, and $1+s$, respectively, where s is the selective advantage for the homozygote of the derived allele and h is the degree of dominance. The program `forwardtraj` simulates trajectories of the derived allele frequency when the age of the derived allele, the selection intensity, and the dominance coefficient are specified. `backwardtraj` simulates trajectories when the current frequency of the derived allele, selection intensity and the dominat coefficient are specified.

Compilation and usage:

To compile the programs, type

```
gcc -o forwardtraj forward.c rand_mt32.c -lm
```

and

```
gcc -o backtraj backward.c rand_mt32.c -lm .
```

To run `forwardtraj`, type

`./forwardtraj` *howmany* `-t` *t_mut* `-s` $4N_0s$ `-h` h

where *howmany* is the number of trajectories to be generated. If *howmany* is set to 3, then the program generates three trajectory files, `traj_0.dat`, `traj_1.dat`, and `traj_2.dat`, in the working directory. *t_mut* after the '-t' switch is the time when the derived allele arises by mutation (in units of $4N_0$). The selection intensity is specified as $4N_0s$ after the '-s' switch, and the degree of dominance is specified after the '-h' switch. To run `backwardtraj`, type

`./backwardtraj` *howmany* `-f` $freq$ `-s` $4N_0s$ `-h` h

where $freq$ is the current frequency of the derived allele.

References

Hudson, R. (1990). Gene genealogies and the coalescent process. In D. Futuyma and J. Antonovics, editors, *Oxford Surveys in Evolutionary Biology*, volume 7, pages 1–44. Oxford University Press.

Kimura, M. and Takahata, N. (1983). Selective constraint in protein polymorphism: study of the effectively neutral mutation model by using an improved pseudosampling method. *Proc Natl Acad Sci USA*, **80**, 1048–52.
